# Stem cell behaviors on periodic arrays of nanopillars analyzed by high-resolution scanning electron microscope images

**DOI:** 10.1186/s42649-020-00046-3

**Published:** 2020-11-17

**Authors:** Jihun Kang, Eun-Hye Kang, Young-Shik Yun, Seungmuk Ji, In-Sik Yun, Jong-Souk Yeo

**Affiliations:** 1 School of Integrated Technology, Yonsei University, 85 Songdogwahak-ro, Yeonsu-gu, Incheon, 21983 South Korea; 2 Yonsei Institute of Convergence Technology, Yonsei University, 85 Songdogwahak-ro, Yeonsu-gu, Incheon, 21983 South Korea; 3 Department of Plastic and Reconstruction Surgery, College of Medicine, Yonsei University, 50-1 Yonsei-ro, Seodaemun-gu, Seoul, 03722 South Korea

**Keywords:** Cell behavior, Human adipose-derived stem cell, Scanning electron microscope, Polyurethane acrylate, Nanopillar

## Abstract

The biocompatible polyurethane acrylate (PUA) nanopillars were fabricated by soft lithography using three different sizes of nanobeads (350, 500, and 1000 nm), and the human adipose-derived stem cells (hASCs) were cultured on the nanopillars. The hASCs and their various behaviors, such as cytoplasmic projections, migration, and morphology, were observed by high resolution images using a scanning electron microscope (SEM). With the accurate analysis by SEM for the controlled sizes of nanopillars, the deflections are observed at pillars fabricated with 350- and 500-nm nanobeads. These high-resolution images could offer crucial information to elucidate the complicated correlations between nanopillars and the cells, such as morphology and cytoplasmic projections.

## Description

Analyzing cell behaviors and controlling them is vital for cell research, and there have been many efforts for developing novel methods and instruments to reveal complex correlations of the cell and its behaviors, and to control them (Stevens and George 2005). Among them, investigating and controlling cell behavior via nanopillars become one of the high-interest topics. So, researchers started to investigate the novel methods for not only measuring cellular traction forces with nanopillars (Tan et al. 2003; Trichet et al. 2012) but also controlling cell behaviors by altering the sizes and pitches of nanopillars (Yun et al. 2020).

However, the high aspect ratio nanopillars could not offer sufficient top surface area for generating focal adhesions, and the proper stiffness of pillars was difficult to provide for cell culture. Herein, by using biocompatible PUA, the novel substrates of different pillar sizes and periodic arrays were fabricated to culture hASCs on the nanopillars in order to understand the mechanotransduction of hASCs and their various behaviors, such as cytoplasmic projections, migration, and morphology.

**Fig. 1:**
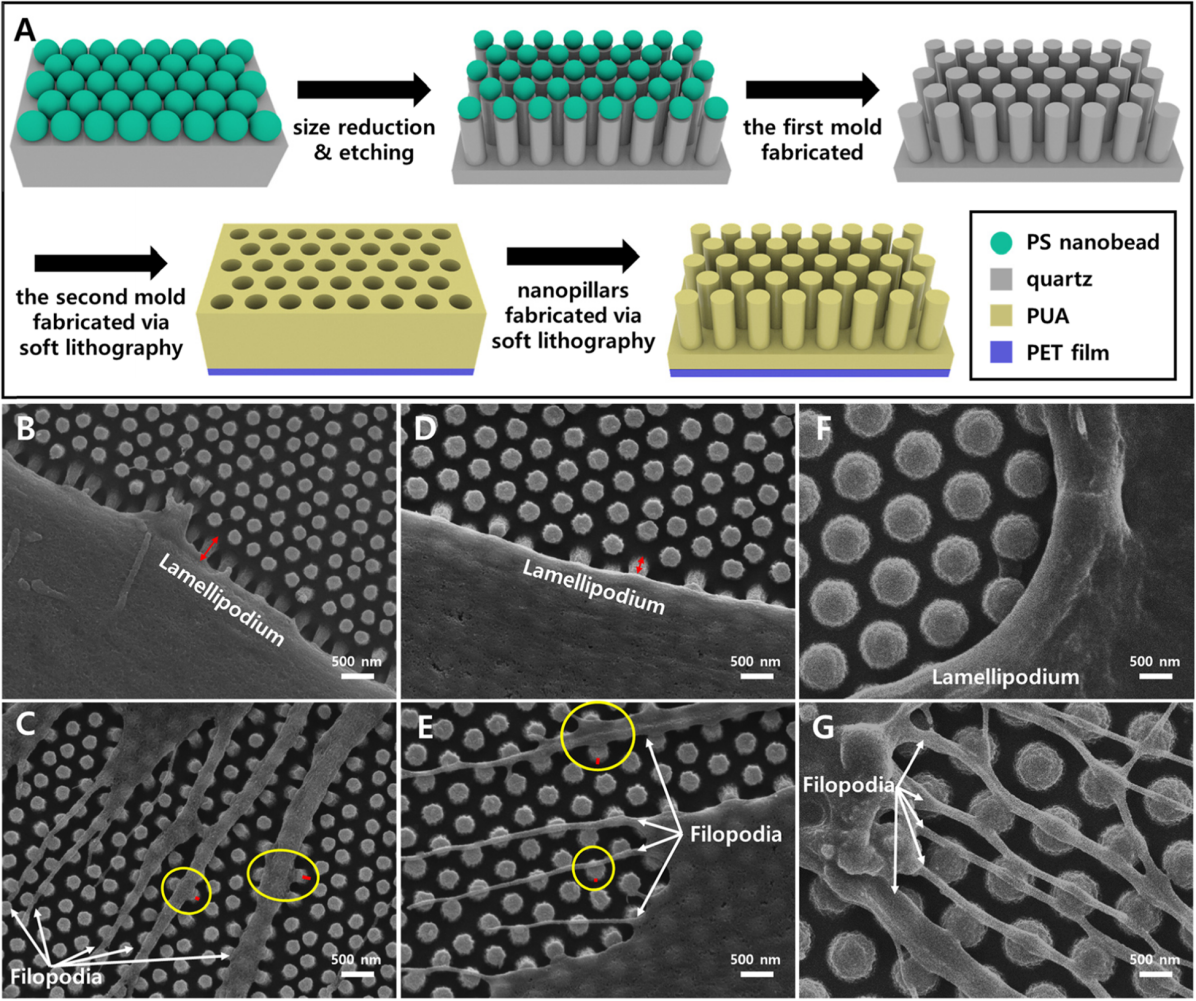
The fabrication process of PUA nanostructures (**a**), and the SEM images of hASCs cultured on the nanopillars fabricated by soft lithography with **b**, **c** 350; **d**, **e** 500; and **f**, **g** 1000 nm nanobeads. **b**, **d**, **f** Interaction of nanopillars with the lamellipodia of a stem cell. **c**, **e**, **g** Interaction of nanopillars with the filopodia of a stem cell. On **b** and **d**, red arrows show the deflection of pillars. On **c** and **e**, the difference of the deflection of pillars is shown with red bars in yellow circles. Unlike nanopillars with 350- and 500-nm nanobeads, the nanopillars fabricated with 1000-nm nanobeads did not show deflection for both of **f** and **g**

As shown in Fig. 1a, to fabricate the nanopillars, 350-, 500-, and 1000-nm polystyrene (PS) nanobeads were used for forming a monolayer on a quartz substrate via self-assembly. Then, the size reduction process was conducted on the nanospheres of the monolayer by oxygen plasma. With the reduced particles, capacitively coupled plasma (CCP) etching was performed to fabricate the first mold. The final mold, composed of nanohole arrays, was fabricated with biocompatible and UV curable PUA via soft lithography.

With the final mold and the soft lithography, various sizes and periodic arrays of PUA nanopillars could be fabricated. The diameters of the PUA pillar are 200, 270, and 710 nm, and the spacings between pillars are 170, 220, and 290 nm, respectively. The heights of pillars are identical to 400 nm. For SEM observation, the specimens were coated with platinum at 40 mA for 40 s (Cressington sputter coater 208HR), and the images were taken at a low accelerating voltage (5 kV) with JEOL JSM-7100F field emission scanning electron microscope.

The cytoplasmic projections, such as filopodia and lamellipodia, exhibit different cellular traction forces qualitatively indicated by the degree of deflections in Fig. 1. As shown in Fig. 1b and d, lamellipodia protruded with more matured focal adhesions and higher cellular traction forces than filopodia (Fig. 1c, e). And the filopodia exerted higher cellular traction forces as their width increases (yellow circles in Fig. 1c, e). However, not only cytoplasmic projections but also other parts of the cell cultured on nanopillars fabricated with 1000-nm nanobeads did not show any deflection of the pillars (Fig. 1f, g). This suggests that the pillars fabricated with 1000-nm nanobeads may alter the cell behaviors, but they are not suitable for observing deflection to reveal the effect of cellular traction forces.

Controlling cell behavior and fate is the most vital for future bioengineering. With the biocompatible PUA nanopillars, the substrate could offer sufficient area and stiffness for maturing focal adhesions and affecting mechanotransduction of hASCs so that various cell behaviors were altered by different pillar sizes and the relative forces exerted by hASCs could be observed easily with the help of high resolution images based on SEM.

## Data Availability

Not applicable. “Please contact the corresponding author for data requests.”
